# Sleep disorders cause Parkinson's disease or the reverse is true: Good GABA good night

**DOI:** 10.1111/cns.14521

**Published:** 2024-03-16

**Authors:** Hayder M. Al‐kuraishy, Ali I. Al‐Gareeb, Ali K. Albuhadily, Yaser Hosny Ali Elewa, Ammar AL‐Farga, Faisal Aqlan, Mahmoud Hosny Zahran, Gaber El‐Saber Batiha

**Affiliations:** ^1^ Department of Clinical Pharmacology and Medicine, College of Medicine Al‐Mustansiriya University Baghdad Iraq; ^2^ Department of Histology and Cytology, Faculty of Veterinary Medicine Zagazig University Zagazig Egypt; ^3^ Faculty of Veterinary Medicine Hokkaido University Sapporo Japan; ^4^ Biochemistry Department, College of Sciences University of Jeddah Jeddah Saudia Arbia; ^5^ Department of Chemistry, College of Sciences Ibb University Ibb Governorate Yemen; ^6^ Internal Medicine Department, Faculty of Medicine Zagazig University Zagazig Egypt; ^7^ Department of Pharmacology and Therapeutics, Faculty of Veterinary Medicine Damanhur University Damanhur Egypt

**Keywords:** GABA pathway, Parkinson's disease, sleep disorders, γ‐aminobutyric acid

## Abstract

**Background:**

Parkinson's disease (PD) is a progressive neurodegenerative brain disease due to degeneration of dopaminergic neurons (DNs) presented with motor and non‐motor symptoms. PD symptoms are developed in response to the disturbance of diverse neurotransmitters including γ‐aminobutyric acid (GABA). GABA has a neuroprotective effect against PD neuropathology by protecting DNs in the substantia nigra pars compacta (SNpc). It has been shown that the degeneration of GABAergic neurons is linked with the degeneration of DNs and the progression of motor and non‐motor PD symptoms. GABA neurotransmission is a necessary pathway for normal sleep patterns, thus deregulation of GABAergic neurotransmission in PD could be the potential cause of sleep disorders in PD.

**Aim:**

Sleep disorders affect GABA neurotransmission leading to memory and cognitive dysfunction in PD. For example, insomnia and short sleep duration are associated with a reduction of brain GABA levels. Moreover, PD‐related disorders including rigidity and nocturia influence sleep patterns leading to fragmented sleep which may also affect PD neuropathology. However, the mechanistic role of GABA in PD neuropathology regarding motor and non‐motor symptoms is not fully elucidated. Therefore, this narrative review aims to clarify the mechanistic role of GABA in PD neuropathology mainly in sleep disorders, and how good GABA improves PD. In addition, this review of published articles tries to elucidate how sleep disorders such as insomnia and REM sleep behavior disorder (RBD) affect PD neuropathology and severity. The present review has many limitations including the paucity of prospective studies and most findings are taken from observational and preclinical studies. GABA involvement in the pathogenesis of PD has been recently discussed by recent studies. Therefore, future prospective studies regarding the use of GABA agonists in the management of PD are suggested to observe their distinct effects on motor and non‐motor symptoms.

**Conclusion:**

There is a bidirectional relationship between the pathogenesis of PD and sleep disorders which might be due to GABA deregulation.

## Graphical abstract

Role of GABA in sleep disorders and Parkinson's disease.

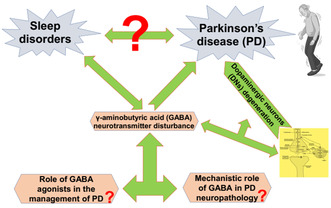

## 1 INTRODUCTION

Parkinson's disease (PD) is one of the most common neurodegenerative disorders that is developed as a result of dopaminergic neuron (DNs) loss in the substantia nigra pars compacta (SNpc).^1^ PD is the subsequent most prevalent neurodegenerative disease, following Alzheimer's disease (AD), that affects 1%–3% of elderly people above the age of 60.^2^ PD has been classified into two types: familial and sporadic PD.^3^ The former type counts for 10‐15% of all PD types.^4^ Numerous genes are involved in PD neuropathology, including alpha‐synuclein (α‐Syn), leucine‐rich repeat kinase 2 (LRRK2), glucocerebrosidase (GBA), vacuolar protein sorting associated protein 35 (VPS35), phosphatase homolog‐induced kinase (PINK1), and Parkinson protein 7 (PAPK7).^5^, ^6^ The interfaces between susceptible genes and environmental factors direct the onset of PD. It has been shown that caffeine affects the expression of the adenosine A2A gene, which is reduced in PD.^7^ Though pesticides affect polymorphisms of the LRRK2, PINK1, and PINK2 genes with succeeding initiation of oxidative stress, mitochondrial dysfunction, and the development of PD.^8^

The risk factors for the development of PD are diverse, including sex, age, and ethnicity.^9^ Noteworthy, old age is a key risk factor for the development of PD that affects onset and disease severity.^10^ The majority of PD cases are developed in 60–65 years, though juvenile PD was described as occurring at ages less than 21 years.^10^ PD prevalence is more in men due to the neuroprotective effect of estrogen in women.^11^ Moreover, PD prevalence has been documented to be more common in western populations than in Asian populations.^12^ Additionally a lower incidence of PD has been reported in the black race due to a higher concentration of neuroprotective neuromelanin in the SNpc.^13^ Furthermore, environmental toxins are implicated in the pathogenesis of PD, notably heavy metals, which prompt PD neuropathology through numerous mechanisms, including oxidative stress and mitochondrial dysfunction, with subsequent synaptic dysfunction and disruption of brain neurotransmission.^14^ In addition, iron is tangled in the pathogenesis of PD through the induction of oxidative stress and mitochondrial dysfunction, with an intensification of α‐synuclein neurotoxicity.^15^ Likewise, contact with lead and manganese stimulates the injury of dopaminergic neurons and the development of PD.^16^, ^17^ Furthermore, prolonged use of psychostimulant agents like methamphetamine and cocaine increases PD risk through inhibition of dopamine transporters in the early stage and degeneration of dopaminergic neurons in the late stage.^18^

**FIGURE 1:**
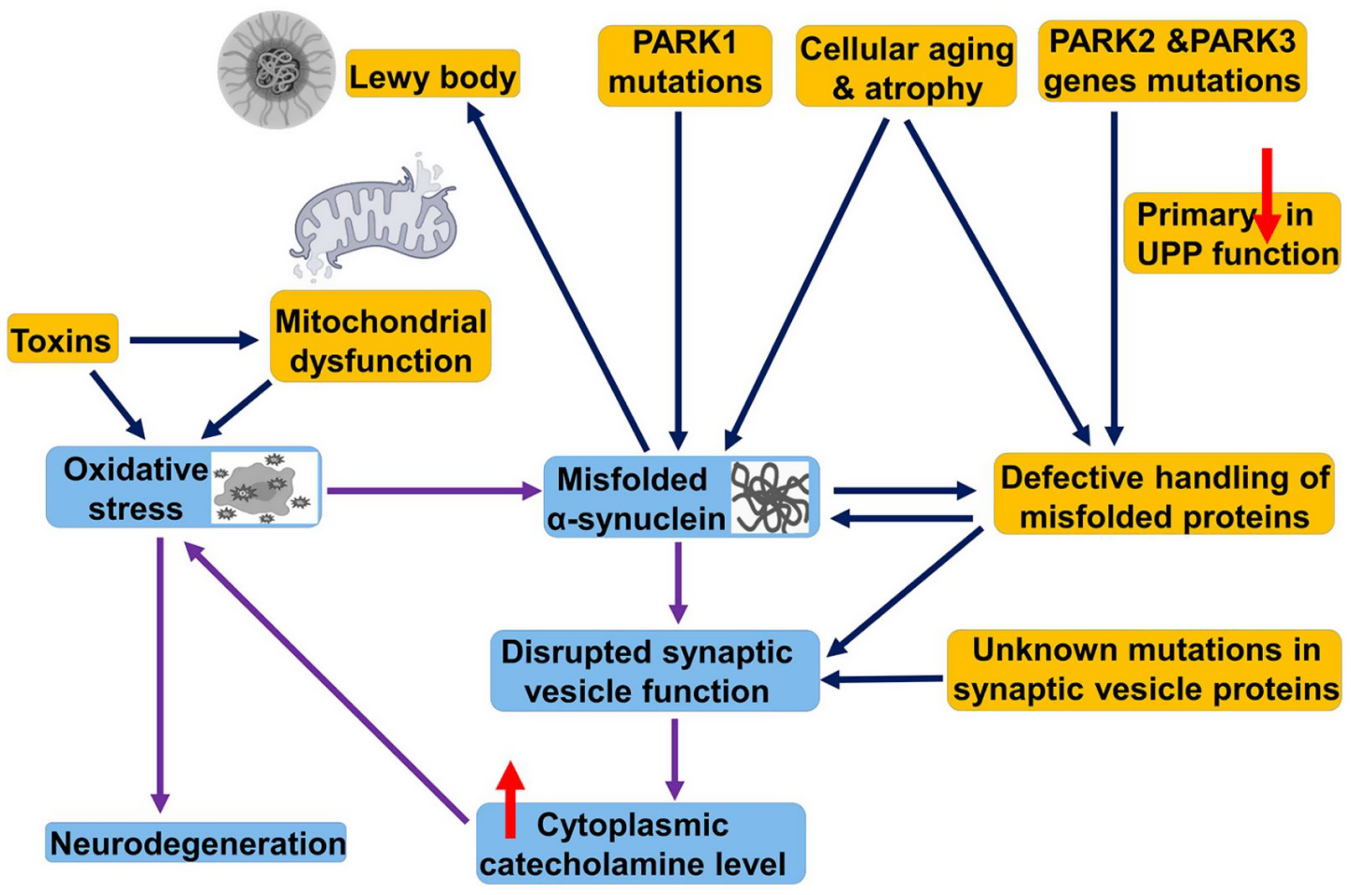
Pathophysiology of Parkinson disease: Oxidative stress and mitochondrial dysfunction, together with genetic mutations, promote the aggregation of misfolded α‐Syn, leading to the formation of Lewy bodies and disruption of synaptic vesicle function with an increase in cytoplasmic catecholamine levels, which, through the induction of oxidative stress, leads to neurodegeneration. PARK2 and PARK3 gene mutations reduce the handling and clearance of misfolded proteins, leading to the accumulation of α‐Syn. Therefore, environmental toxins and genetic factors contribute to the pathogenesis of PD.

The pathophysiology of PD has not been recognized exactly; nevertheless, some neuropathological features are discovered (Figure 1).

The main pathological features in PD neuropathology are neuronal cell death in the SNpc due to the deposition of α‐Syn.^19^ Noteworthy α‐Syn is typically found in the central nervous system (CNS) at presynaptic site and plays a critical role in the release of neurotransmitters, including dopamine.^20^ Genetic variations together, with environmental toxins, trigger misfolding and aggregation of α‐Syn and the formation of Lewy bodies in the neurons.^21^ These neuropathological changes prompt the death of neuronal cells and astrocytes with the forceful activation of microglia in the SNpc.^22^ Neuronal degeneration induced by α‐Syn is interceded by a direct toxic effect or indirectly through the induction of mitochondrial dysfunction, proteasomal and lysosomal dysfunctions leading to protein aggregation and the induction of oxidative stress with subsequent neuronal cell death.^23^ Rendering to Braak staging, PD neuropathology starts in the olfactory bulb and medulla before affecting the SNpc, suggesting a prion‐like disease.^24^ The major neuronal tracts connecting basal ganglia to the other brain regions are the orbitofrontal, limbic, associative, oculomotor, and motor tracts that are affected in PD neuropathology, leading to motor and non‐motor symptoms.^25^ Dopamine neurotransmitter released from the DNs is responsible for regulation of motor activity, though a low dopamine level is correlated with hypokinesia, while increasing dopamine activity leads to inappropriate motor activity called dyskinesia.^26^

The fundamental clinical features of PD are motor symptoms, including bradykinesia, resting tremor, and rigidity.^27^ Motor symptoms are developed when more than 70% of DNs in the SNpc are lost.^28^ Nevertheless, non‐motor symptoms, including autonomic dysfunction, constipation, anosmia, sleep disorders, and cognitive dysfunction, develop prior to the motor symptoms.^29^ It has been shown that PD symptoms develop in response to disturbances of different neurotransmitters, including acetylcholine (Ach), dopamine, and γ‐aminobutyric acid (GABA).^30^ As GABA strongly affects the early stages of sleep, the effect of GABA on sleep may be connected to its levels in the blood. A randomized, single‐blind, placebo‐controlled crossover‐designed study showed that oral GABA administration significantly shortened sleep latency and increased the total non‐rapid eye movement (non‐REM) sleep time.^31^ Besides, within the CNS, the GABA mechanism stabilizes neuronal activity both at cellular and systemic levels. The decline in the GABA control initiates several cascading processes, leading to both weakened protective barriers and accumulations of intracellular calcium and Lewy bodies.^32^ Thus, the original description of PD as due to the selective damage of DNs in the mesencephalon should be updated into the concept of a severe multi‐systemic neurodegenerative disorder of the nervous system, whose clinical symptoms reflect the localization and progression of the most advanced GABA pathology.^32^ A future and more complete therapeutic approach to PD should be aimed first at slowing the progression of GABA functional decline. However, little is recognized about the role of GABA in PD neuropathology, mainly sleep disorders. Therefore, this review aimed to clarify the potential role of GABA in PD regarding sleep disorders.^33^

## 2 GABA OVERVIEW

**FIGURE 2:**
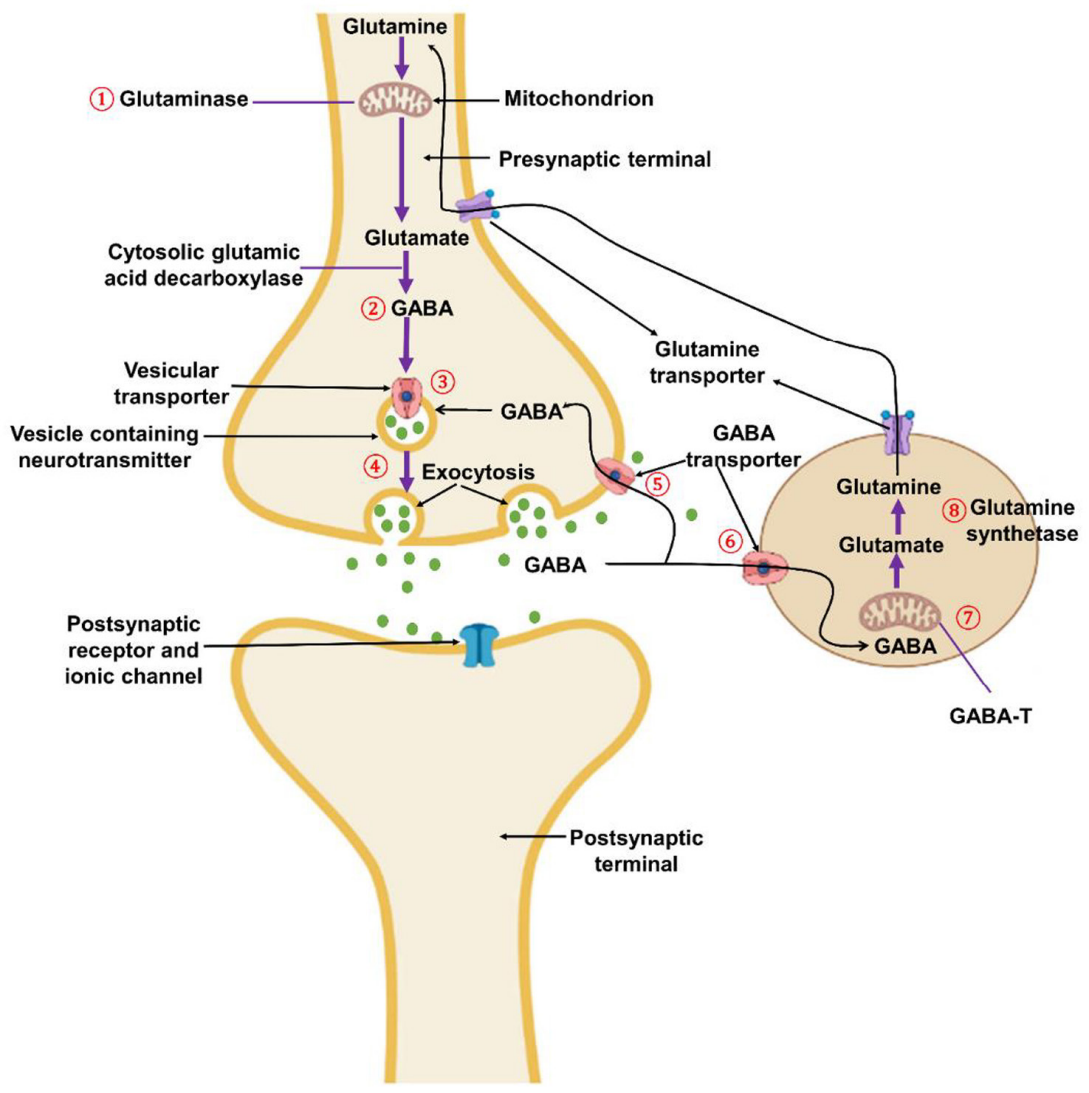
GABAergic neurotransmission: At the presynaptic terminal, glutamine by mitochondrial glutaminase is converted to glutamate, which by cytosolic glutamic acid decarboxylase is converted to GABA, which is released by exocytosis. In the synaptic cleft, GABA either reuptakes to the presynaptic terminal by the GABA transporter or is metabolized by the GABA transaminase to glutamine, which, via the GABA transporter, is recycled again to the presynaptic to form GABA. GABA acts on the GABA receptors at postsynaptic terminals.

GABA is considered a multi‐functional molecule in the CNS, peripheral nervous system, and non‐neuronal tissues.^34^ GABA is an inhibitory neurotransmitter widely expressed in the CNS.^35^ GABA acts on the GABA receptors, comprising GABA_A_, GABA_B,_ and GABA_C_.^35^ GABA_B_ is a G‐protein metabotropic receptor, while GABA_A_ and GABA_C_ are Cl‐gated channels^36^ (Figure 2).

The GABA receptor is composed of three central subunits, which are α, β, and γ, in a heteromeric or homomeric fashion.^36^ GABA regulates neuronal activity through the opening of K^+^ or inhibiting of Ca^2+^ via the G‐protein signaling pathway.^37^ GABA, by the action of glutamic acid decarboxylase (GAD), is synthesized from glutamate; the formed GABA is transported to the presynaptic terminals and stored in specialized vehicles.^38^ GAD is widely expressed in the CNS inhibitory neurons and associated with GABA neurons. Dysregulation of GAD is linked with the development of schizophrenia and epilepsy.^38^, ^39^ Depolarization of presynaptic GABAergic neurons activates the release of GABA into synaptic space with subsequent stimulation of post‐GABA receptors and post‐synaptic inhibition.^40^ In addition, GABA from the synaptic cleft may leak outside and activate extra‐synaptic GABA receptors, causing tonic inhibition.^41^ Particularly, GABA_A_ in the dorsal horn is found in both presynaptic and postsynaptic neurons, mediating presynaptic inhibition and primary afferent depolarization, correspondingly.^42^ Extra‐synaptic α5‐GABA_A_ on the proprioceptive afferent neurons leads to tonic depolarization of the spinal cord via modulation of Na^2+^ channels.^43^ Furthermore, GABAergic neurons are excitatory at prenatal and postnatal periods; nevertheless, these neurons endure developmental changes from excitatory to inhibitory.^44^, ^45^ Of interest, the polarity of GABA neurons to inhibitory functions is frequently mediated by the K^+^–Cl^−^ co‐transporter (KCC2).^44^ Oxytocin plays a critical role in the transition of GABAergic neurons to inhibitory functions through modulation of KCC2.^46^, ^47^, ^48^ In the absence of oxytocin, the activity of KCC2 is abridged with an increase in hyperexcitability and related disorders like epilepsy and neurodevelopmental disorders.^46^ In addition, the expression of GABA_A_ receptors is controlled by the allopregnanolone steroid, which exerts positive and negative impacts on acute and chronic effects correspondingly on the expression of GABA_A_ receptors and the progression of dementia.^49^ Moreover, dysfunction of GABAergic neurons in the frontal lobe increases the risk of the development of fronto‐temporal dementia.^50^

These outcomes indicated the potential role of GABAergic neurons in the regulation of neuronal inhibition and the prevention of neuronal hyperexcitability.

## 3 GABA PATHWAY AND NEURODEGENERATIVE DISORDERS

GABAergic neurons are involved in the direct action of memory and learning, hallmark variables in the development of AD.^50^ Neuronal injury by the deposition of Aβ induces synaptic dysfunction with subsequent disruption of excitatory/inhibitory circuits, causing cognitive deficits.^51^ Interaction of Aβ with the neurotransmission network in brain areas linked to memory, such as the hippocampus and amygdaloid complex, leads to memory impairment and cognitive dysfunction.^52^ Regulation of cognitive function is mostly fine‐tuning between the excitatory neurotransmitters and GABA inhibitory systems.^53^ Hippocampal and cortical functions are mainly dependent on the GABA inhibitory function to maintain the activity of synaptic plasticity.^54^ Chiefly, hippocampus GABA inhibitory neurons represent 10–15% of total brain inhibitory neurons.^55^ Confirmations from accumulating studies show that GABAergic neurons are extremely dysregulated and intricate in the pathogenesis of AD.^56^ In AD, excitatory neurotransmitters are involved in the pathogenesis of Aβ and tau deposition due to the reduction of the protective GABA inhibitory function.^57^ Accordingly, disruption of the inhibitory/excitatory axis in the brain could be a possible mechanism for the development of seizures in AD patients.^58^

Certainly, GABAergic neurons are exceedingly disturbed in many neurodegenerative diseases, including AD, and could be a therapeutic target in the management of these disorders.^59^ An experimental study illustrated that the density of GABAergic neurons was reduced adjacent to Aβ plaques,^60^ suggesting toxic effects of Aβ plaques on the viability of GABAergic neurons. Short and snappy, Aβ plaques are more toxic to the hippocampal GABAergic neurons in the AD mouse model, with a significant reduction in GAD activity.^61^ The in vitro neurotoxicity of Aβ against GABAergic neurons was documented.^62^ Aβ triggers perforation of the cell membrane with increasing Ca^2+^ efflux in the hippocampal GABAergic neurons with disturbances of excitatory‐inhibitory synaptic function.^63^ The concentration of GABA is highly reduced in the temporal cortex and other brain regions in AD patients.^64^ Likewise, GABA levels are also reduced in the cerebrospinal fluid (CSF) in AD.^65^

Furthermore, deficiency of GABA is associated with the development of Huntington chorea and other neurodegenerative disorders, as well as neuropsychiatric disorders like panic, depression, and anxiety.^66^ Similarly, inflammatory reactions in multiple sclerosis (MS), mainly IL‐1β, inhibit GABA function with significant alterations of the GABA pathway.^67^, ^68^, ^69^ A case–controlled study observed that CSF GABA was reduced in MS patients compared to controls.^70^ Changes in GABAergic neurons and reductions in GABA levels are also correlated with the severity of cognitive impairment in MS patients.^70^ In relapsing–remitting MS (RRMS) patients, GABA+ levels were lower in the posterior cingulate cortex and left hippocampus compared with controls; decreased GABA+ in the posterior cingulate cortex and left hippocampus was associated with specific cognitive functions.^70^ Thus, abnormalities of the GABAergic system may be present in the pathogenesis of RRMS and suggest a potential link between regional GABA levels and cognitive impairment in patients with RRMS. Moreover, in amyotrophic lateral sclerosis (ALS), GABA pathway is exceedingly deregulated, leading to cortical hyperexcitability.^71^ A cross‐sectional study revealed that GABAergic neurons were reduced in ALS patients, causing more severe motor alterations.^72^ Glutamate is a chemical mediator in the brain that stimulates motor neurons. Glutamate overproduction is probably responsible for motor neuron damage in ALS. The molecule GABA acts to lessen the effects of glutamate. Medicines that increase GABA activity (GABA modulators), such as gabapentin and baclofen, are possible treatments for ALS.^72^ According to high‐quality evidence, gabapentin is not effective in treating ALS. It does not extend survival, slow the rate of decline of muscle strength and respiratory function, and, based on moderate‐quality evidence, probably does not improve quality of life or slow monthly decline in the ALS.^72^

These findings anticipated that the GABA pathway is vastly disturbed in patients with neurodegenerative disorders, and targeting this pathway could be a potential therapeutic strategy against the development and progression of neurodegenerative disorders.

## 4 GABA PATHWAY AND PD NEUROPATHOLOGY

It has been shown that GABA has a protective effect against the degeneration of DNs in SNpc. GABA transporter 1 inhibitor tiagabine attenuates MPTP‐ and LPS‐induced dopaminergic toxicity, inhibits microglial activation in vivo, and improves motor behavior in PD mice.^73^ Blockade of microglial activation conferred beneficial effects in the MPTP mouse model of PD.^74^ Muscimol and baclofen also blocked microglial activation in the LPS model of PD.^73^ The underlying mechanisms by which GABAergic drugs protect DNs are through inhibition of the expression of the pro‐inflammatory signaling pathway.^73^ DA neurons utilize an alternative GABA synthesis pathway to support functional GABAergic neurotransmission.^75^ Thus, GABA alternative synthesis may represent a more fundamental mechanism employed by broader classes of GABAergic neurons.

Disturbance of GABAergic neurons had been observed in the basal ganglia postmortem of PD.^70^ Hypokinetic disorders as in PD are thought to be associated with excessive tonic and phasic inhibition of the output from the basal ganglia to the thalamus. The concentrations of the excitatory neurotransmitters aspartate/glutamate and of the inhibitory neurotransmitter GABA in 18 relevant regions of the thalamocortical circuits of the basal ganglia of patients who have Parkinsonian symptoms and compared them with controls of individuals who have died without any history of neurological or psychiatric disorders and had no neuropathological abnormalities are diminished by 36% in the centromedial thalamus compared to control values.^76^ Also, dopamine is co‐released with GABA from DNs, independent of vesicular GABA transporters.^77^ The release of GABA necessitates the activation of vesicular monoamine transporter 2 (VMAT2), which is also a transporter of dopamine.^77^ The expression of VMAT2 on the GABAergic neurons plays a vital role in the release of GABA.^78^ In addition, DNs in the SNpc inhibit the striatum via presynaptic activation of GABA receptors.^79^ Increasing striatal input by deficiency of GABA leads to the development of bradykinesia in PD.^80^ Depletion of DN receptor 2 (D2R) from the indirect pathway induces severe motor impairment in mice by reducing GABAergic neurotransmission.^80^ There was a robust enhancement of GABAergic transmission and a reduction of in vivo firing in striatal and pallidal neurons. Mimicking D2R signaling in indirect‐pathway medium spiny neurons may restore the level of tonic GABAergic transmission and rescue the motor deficit. Thus, D2R activation regulates motor output by constraining the strength of GABAergic transmission.^80^ These findings suggest an intricate interaction between dopamine and GABA. Therefore, degeneration of DNs is associated with a reduction of GABA in PD. Hyperpolarization of GABAergic neurons regulates presynaptic neurotransmission and prevents neuronal hyperexcitability through the maintenance of Ca^2+^ homeostasis.^81^ This effect attenuates Ca^2+^‐induced DN injury. It is now generally established that α‐Syn can be released in the extracellular space, even though the mechanism of its release is still unclear. In addition, pathology‐related aggregated species of α‐Syn have been shown to propagate between neurons in synaptically connected areas of the brain, thereby assisting the spreading of pathology in healthy neighboring neuronal cells.^82^ Notably, DNs are highly vulnerable to the neurotoxic effects of α‐Syn due to the higher expression of Ca^2+^ voltage‐gated channels, which increase the release of α‐Syn both in vitro and in vivo, with subsequent aggregation and the development of synucleinopathy.^82^ Consequently, regulation of Ca^2+^ voltage‐gated channels by GABA may prevent Ca^2+^‐induced excitotoxicity, oxidative stress, mitochondrial dysfunction, and the development of PD. Thus, reduction of GABA promotes oxidative stress and mitochondrial dysfunction, which are linked with PD neuropathology.^83^ Hence, the restoration of GABA activity by GABA agonists can reduce motor symptoms in the PD model.^84^ GABA agonists can protect DNs and striatal terminals from oxidative stress in 6‐hydroxydopamine (6‐OHDA)‐induced PD in rats.^84^ Improvement of inhibitory GABAergic neurons by GABA agonist bumetanide may reduce the severity of motor symptoms in PD.^85^

An experimental study showed that dysregulation of GABAergic neurons in the SNpc leads to abnormal neuronal firing.^86^ A recent study established that induction of GABAergic neuron generation by astrocyte reprogramming improves motor symptoms in experimental PD.^87^ Short‐interval intracortical inhibition is mediated by GABA_A_ receptors, and long‐interval intracortical inhibition is mediated by GABA_B_ receptors.^88^ In PD, presynaptic inhibition is decreased, leading to abnormal neuronal circuits. A case‐control study illustrated that pre‐synaptic inhibition in the motor cortex is reduced in PD patients, and this may clarify the non‐DN feature of PD.^88^ However, thalamocortical GABAergic neuron activity is increased in PD.^89^ Motor cortex GABA level is inversely correlated with PD disease; therefore, GABA depletion may participate in the development of motor symptoms.^89^ A case‐control study that included 60 PD patients with dopamine‐resistant tremor (*n* = 17), dopamine‐responsive tremor (*n* = 23), or no tremor (*n* = 20), and healthy controls (*n* = 22) showed that motor cortex GABA levels measured by resonance spectroscopy were inversely correlated with disease severity, particularly rigidity and tremor.^89^ Thus, cortical GABA plays a beneficial rather than a detrimental role in PD, and GABA reduction may increase motor symptoms. Furthermore, a GABAergic deficit in the brainstem may contribute to the PD neuropathology.^90^ A case‐control study involved 18 PD patients and 18 healthy control subjects and observed that GABAergic neuron activity in the upper brainstem, as measured by resonance spectroscopy, was reduced in PD patients compared to the controls.^90^ Many circuits involving dopaminergic projections from the midbrain also include GABAergic inhibitory projections. For example, those to the striatum are mirrored by GABAergic projections behind the midbrain and SNpc. Altered dopaminergic activity in PD substantially impacts the GABAergic circuitry of the SNpc and impacts the excitation/inhibition balance in the cortex.^91^ Numerous brainstem nuclei are engaged in fundamental homeostatic mechanisms, including gastrointestinal regulation, pain perception, mood control, and sleep‐wake cycles,^92^ all of which are impacted by PD. Hence, a reduction of brainstem GABA could be involved in the development of non‐motor symptoms in PD. These findings indicated that GABA has a protective role against PD neuropathology. A reduction in GABA signaling is implicated in the pathogenesis of PD.

## 5 GABA PATHWAY AND MANIFESTATIONS OF PD

GABAergic signaling controls a wide range of physiological functions, including cognition, information processing, and sensory perception.^93^ The GABA pathway has a vital role in controlling inhibitory tone in the globus pallidus (GP), SNpc, and thalamus to prevent excessive stimulation of the cerebral cortex.^87^ Deregulation of the GABA pathway in PD triggers neuronal hyperexcitability, causing dyskinesia or bradykinesia.^88^ In addition, deregulation of the GABA pathway may be involved in the development and progression of motor and non‐motor manifestations in PD. The mechanism of motor dysfunction in PD is well‐defined due to dysfunction of DNs; however, alteration of GABA is also concerned in PD neuropathology.^77^, ^94^

### 5.1 Motor manifestations

Cardinal motor dysfunction in PD is developed due to the degeneration of DNs in the SNpc. Nevertheless, in advanced PD, dyskinesia and motor fluctuation are developed due to degeneration of non‐DN pathways.^77^ Interestingly, increasing the activity of GABAergic neurons may improve motor symptoms in PD. GABA_A_ agonist zolpidem has an extraordinary beneficial effect on reducing dyskinesia even after a single dose in PD patients.^95^ Zolpidem has a peculiar effect on movement disorders in PD patients; other GABA_A_‐agonist hypnotics like zopiclone and triazolam produced no beneficial motor effects in women with PD.^95^ PD treatments that focus on the dopaminergic system alone are unable to alleviate both motor and non‐motor symptoms, particularly those that develop in the early stages of the disease. The development of agents that interact with several of the affected neurotransmission systems could prove invaluable for the treatment of this disease.^94^ High‐frequency stimulation (HFS) of the subthalamic nucleus proves to be an efficient treatment for alleviating motor symptoms in PD by increasing GABA release in the SNpc of experimental rats.^96^ A decline in the tonic GABA inhibitory activity of the basal ganglia results in increasing co‐activation of different competitive motor programs, which causes co‐activation of a variety of muscle groups, including co‐contractions of agonist and antagonist muscles and progressive stiffness, which leads to progressive changes in posture and a rigid gait.^97^ GABA compounds that cross the BBB increase GABA activity and improve muscle stiffness caused by a lack of GABAergic tone. Also, the use of GABA‐producing transplants for the recovery of function in the rat PD model introduces a novel concept of therapeutic intervention in PD.^98^ Furthermore, the protective effects of glial cell‐derived neurotrophic factor (GDNF) for midbrain DNs are observed only when the GDNF is delivered into the GABAergic striatum but not directly to the SNpc.^99^ The input GABA neurons of the SNpc have a high threshold for activation and are essentially silent. An increasing threshold of striatal input due to GABA deficiency would be manifested in bradykinesia and hypokinesia. In this case, GABA deficiency at the striatal input to the SNpc would require increased dopamine input. GABA striatal spiny neurons forming an input system to the SNpc are only activated during motor activity and they do not seem to degenerate.^98^ These outcomes suggest that degeneration of GABAergic neurons is implicated in the development and progression of motor symptoms in PD.

### 5.2 Non‐motor manifestations

It has been shown that non‐motor manifestations of PD, such as cognitive dysfunction, sleep disorders, olfactory dysfunction, gastrointestinal disorders, and visual disturbances, are the major source of PD burden.^100^ Non‐motor manifestations precede the development and progression of motor symptoms by years due to GABAergic dysfunction.^100^

Furthermore, cognitive dysfunction is frequently associated with PD in about 20%–25%.^101^ It has been reported that PD patients have a greater risk of developing dementia and cognitive dysfunction compared to the controls.^101^, ^102^ Cognitive dysfunction in PD may expand due to the deregulation of various neurotransmitters like Ach and dopamine in the fronto‐striatal pathway.^103^ Cognitive dysfunction in PD is correlated with both motor and non‐motor symptoms.^104^ Furthermore, somatostatin‐expressing GABAergic neurons have excitatory effects on the cortical circuits regulating neuronal activity.^105^ Therefore, dysfunction of GABAergic neurons is associated with the development of cognitive dysfunction. It has been presented that GAD expression is reduced in PD patients, leading to a reduction in the neuronal synthesis and release of GABA.^106^ Findings from the postmortem study showed that GAD67 expression was decreased in the prefrontal cortex of PD patients compared to controls.^106^ Besides, GABA activity is decreased in PD patients during cognitive stress and stimulation.^107^, ^108^ Furthermore, blunted GABA responses to dopamine agonists in PD patients lead to behavioral and cognitive abnormalities.^109^ These annotations propose that GABAergic dysfunction in PD is associated with the progression of cognitive dysfunction.

Indeed, olfactory disorders are frequent in PD, and more than 90% of PD patients have this disorder.^110^, ^111^ A study demonstrated that the volume of the olfactory bulb measured by MRI volumetric measurement was smaller in PD patients (*n* = 25) compared to the matched controls (*n* = 40).^110^ A postmortem study discovered that the volume of the olfactory bulb in PD patients was smaller compared to healthy controls.^112^ Besides, microstructural changes in the olfactory bulb are correlated with dysfunction of DNs in the putamen.^112^ Olfactory dysfunction in PD is correlated with neuronal loss and structural changes in the nucleus basalis, raphe nuclei, and locus coeruleus.^111^ Herein, these neuroanatomical changes propose the participation of serotonergic, noradrenergic, and cholinergic in olfactory dysfunction.^111^ GABAergic neurons in the olfactory pathway regulate odor perception and sensitivity.^113^ Particularly, the development of aberrant GABAergic neurons is associated with olfactory dysfunction in AD.^114^ Thus, dysfunction of GABAergic neurons in PD could be the main mechanism for the development of olfactory dysfunction. In this state, potentiating GABAergic neurons may alleviate olfactory dysfunction in PD. An experimental study demonstrated that the GABA agonist muscimol mitigates olfactory dysfunction in mice.^115^ In addition, the development of olfactory dysfunction in PD may be linked to dementia.^116^ In addition, deficits of other neurotransmitter systems such as serotonergic, noradrenergic, and cholinergic projections to the olfactory bulb are potentially associated with olfactory dysfunctions in PD. Moreover, the pronounced olfactory deficits in PD are associated with a higher risk for developing dementia, which strengthens the use of odor tests as possible early diagnostic methods.^116^ As a result, early appreciation and management of olfactory dysfunction may abrogate the development of PD‐related dementia.

Furthermore, dysregulation of the GABAergic pathway is connected with the development of neuropsychiatric disorders like depression and anxiety.^117^ Of note, somatostatin‐expressing GABAergic neurons are reduced in PD patients with parkin mutations.^118^ In the CNS, somatostatin is extremely co‐localized with GABAergic neurons; it acts as a neuromodulator, or co‐neurotransmitter, that controls the function and activity of these neurons; thus, the CSF somatostatin level reflects the density and activity of GABAergic neurons.^119^, ^120^ CSF somatostatin levels were reduced in PD.^121^ Somatostatin‐like immunoreactivity in the CSF of 35 aged PD patients was highly reduced.^121^ A systematic review and meta‐analysis demonstrated that PD neuropathology is linked to functional and structural changes in the neuronal circuits concerned with the pathogenesis of anxiety and motor deficits.^122^ Similarly, a systematic review and meta‐analysis revealed that depression is found in 38% of PD patients and is more connected with female sex and the GBA1 mutation.^122^ In addition, depression is regarded as an independent non‐motor symptom of PD that appears in the early stages and continues throughout the disease duration.^123^ Dysfunction of the GABAergic pathway is associated with the development of depressive disorders.^124^ A case‐controlled study showed that CSF GABA was low in depressed patients compared to the controls.^125^ These results advocate that dysfunction of the GABAergic pathway is allied with the development of depression and anxiety in PD.

Moreover, GABA concentration was reduced in the occipital cortex, leading to visual hallucinations. A cohort study that involved 39 PD patients – 19 with hallucination and 17 without hallucination – showed that GABA concentration measured by magnetic resonance spectroscopy was reduced in the occipital cortex and correlated with excitability in PD patients with hallucinations.^126^ It has been shown that PD patients had visual disturbances with abnormal color vision in the late stage due to alteration of retinal GABAergic neurons.^127^ As well, increasing the number of retinal GABAergic neurons by GABA agonists also induces visual disturbances.^128^ Therefore, an optimal GABA level is crucial for normal visual accuracy and discrimination. GABAergic neurons regulate visual perception, and deregulation of GABAergic neurons is associated with the development of visual disturbance in PD.^129^ Visual disturbances and retinal abnormalities are observed in PD patients and animals due to the deposition of α‐Syn in the retina.^130^

In PD, gastrointestinal (GIT) disturbances, including constipation, gastroparesis, nausea, vomiting and hypersalivation, are common due to dysfunction of the enteric nervous system (ENS) and degeneration of the vagus nucleus in the brainstem.^131^, ^132^ GABAergic pathway regulates intestinal motility and peristaltic reflex.^133^ Of interest, all types of GABA receptors are exceedingly expressed in the GIT and are concerned with the regulation of excitatory and inhibitory signaling in the ENS as well as GIT inflammation.^133^ Therefore, GABA receptor agonists can improve GIT disturbances and inflammation in mice.^133^ Moreover, GABA at low concentrations exerts an inhibitory effect, while higher concentrations lead to an inhibitory effect on GIT peristaltic activity.^134^ GABA_A_ agonist muscimol induces an excitatory effect on the GIT peristaltic activity that is blocked by GABA_A_ antagonist bicuculline.^134^ Consequently, GABA is regarded as a modulator of colonic peristalsis through modulation of Ach release from enteric neurons.^134^ These remarks indicated that dysfunction of the GABAergic pathway in PD is implicated in the GIT disturbances.

In sum, deregulation of the GABAergic pathway in PD could be implicated in the development and progression of motor and non‐motor symptoms in PD, and augmentation of this pathway by GABA agonists might be effective in the management of PD.

## 6 GABA PATHWAY AND SLEEP DISORDERS

Sleep disorders are associated with dysfunction of the GABA pathway.^135^ Subclinical insomnia and short sleep duration are associated with a reduction in brain GABA level. Besides, reduction of prefrontal GABA is linked with memory and cognitive dysfunction.^135^ A cohort study involving 166 subjects with short sleep duration revealed that brain GABA levels, measured by magnetic resonance spectroscopy, were low in subjects with short sleep duration and associated with impairment of working memory.^135^ Over‐activation of GABA in the amygdala is linked with the development of cataplexy.^136^ SNpc has dopaminergic and GABAergic neurons, which project to and gain inputs from the locus coeruleus (LC), which regulates REM‐ON and REM‐OFF neurons. GABAergic neurons prompt the induction of REM sleep.^137^ As well, GABAergic neurons in the ventral tegmental area control NREM sleep.^138^ These findings pointed out that GABA has an important role in normal sleep and that dysfunction of GABAergic neurons is linked with the development of sleep disorders.

It has been estimated that 20% of all brain neurons are GABAergic types, which regulate the basic and integrated sleep‐waking process.^139^ Inhibitory processes have long been considered crucial for brain functioning. While early studies focused on GABAA receptor influences, nowadays research has expanded our knowledge of the inhibitory processes that specifically regulate waking and sleep by including GABA_B_ and the more recently discovered ρ‐containing GABA_A_ (GABA_C_) receptors as well. The main results have shown that both GABA_B_ and GABA_C_ receptor antagonists increase waking and decrease slow‐wave sleep. In contrast, while GABA_B_ receptor antagonists increase REM sleep, GABA_C_ receptor antagonists inhibit it.^139^ GABA receptors affect sleep, but to different extents. In general, GABA_A_ agonists maintain sleep by reducing REM sleep and promoting high‐frequency wave sleep.^140^ However, GABA_A_‐agonist drugs have different effects on the sleep stages.^140^ GABA_B_ agonists like baclofen promote sleep by enhancing REM and NREM sleep.^141^ However, GABA_C_ agonists did not affect REM sleep.^142^ Expression of GABA receptors in relation to pathophysiological conditions may affect sleep patterns. For example, Kantrowitz and his colleagues showed defect in the expression of GABA_B_ was linked with sleep disorders in schizophrenia.^143^ Activation of the extra‐synaptic GABA_A_ receptor by gaboxadol improves sleep onset, pattern, and duration.^144^ However, due to the development of severe adverse effects like disorientation and hallucination, gaboxadol failed to pass the phase III clinical trial.^145^ An experimental study observed that dysfunction of the extra‐synaptic GABA_A_ receptor was linked with the initiation and progression of AD.^43^ The extra‐synaptic GABA_A_ receptor plays a crucial role in mediating the action of hypnotic drugs, alcohol, anesthetic agents, and action of neurosteroids.^146^ Disruption of the extra‐synaptic GABA_A_ receptor pathway, as in PD and epilepsy, may explain the development of sleep disorders in these conditions.^146^ These observations propose that dysregulation of synaptic and extra‐synaptic GABA in various brain diseases is connected with the development of sleep disorders.

## 7 PD AND SLEEP DISORDERS

Sleep disorders are one of the most common non‐motor complications of PD and increase in frequency with advancing disease. The causes of sleep disturbance in PD are numerous, and many patients may have several factors that contribute. These disorders can be broadly classified into those that involve nocturnal sleep and daytime manifestations such as excessive daytime sleepiness.^147^ Some sleep disorders, in particular REM sleep behavior disorder (RBD) and excessive daytime sleepiness (EDS), may arise as a primary manifestation of PD, reflecting the anatomic areas affected by the neurodegenerative process. Appropriate diagnosis of the sleep disturbance affecting a PD patient can lead to specific treatments that can consolidate nocturnal sleep and enhance daytime alertness.^147^ PD neuropathology is linked with sleep disorders, which were reported to be up to 98% in PD patients.^147^ Sleep disorders like restless leg syndrome, insomnia, daytime sleepiness, sleep fragmentation, and RBD are frequently developed in the early stages of PD.^148^ Besides, sleep disorders are adversely affecting the cognitive function of PD patients.^149^ A meta‐analysis and systematic review showed that sleep disorders, mainly RBD, are associated with cognitive dysfunction.^113^ Sleep disorders in PD develop due to the reduced activity of GABAergic neurons^150^; therefore, activation of the GABAergic pathway by benzodiazepines like nitrazepam could be effective in the management of sleep disorders in PD.^151^ Zolpidem is effective for insomnia in PD patients through modulation of the GABAergic pathway; it decreases latency for NREM sleep.^152^ The presence of RBD in prodromal PD is linked with severe motor and non‐motor symptoms, suggesting a disease‐modifying effect of this parasomnia. Imbalance between excitatory and inhibitory neuronal circuits, together with inflammatory changes and impairment of brain oxygenation, could be a proposed mechanism for sleep disorders in PD.^152^ One of the most devastating nonmotor manifestations of PD is dementia. There are few established predictors of dementia in PD. In numerous cross‐sectional studies, patients with REM and RBD have increased cognitive impairment on neuropsychological testing.^153^ A cross‐sectional study involving 61 PD patients revealed a significant association between RBD and dementia risk.^153^ RBD at baseline also predicted the new development of hallucinations and cognitive fluctuations. Thus, RBD was related to an augmented risk of dementia. This indicates that RBD may be a marker of a comparatively diffuse, complex subtype of PD.^153^ In addition, RBD increases the risk of developing neuropsychiatric disorders.^154^ A case‐controlled study included 65 PD patients and 33 healthy controls and observed that 46% of PD patients with RBD were associated with apathy and depression.^154^ Of note, sleep disturbances, mainly non‐apnea sleep disorders, are implicated in the progression and development of PD. A retrospective study revealed that sleep disorders are an independent risk factor for PD.^155^ Also, chronic insomnia and long sleep duration augment PD risk, as confirmed by a previous cohort study.^156^ Moreover, PD‐related disorders like rigidity and nocturia affect sleep patterns, leading to fragmented sleep, which may also affect PD neuropathology.^157^

**FIGURE 3:**
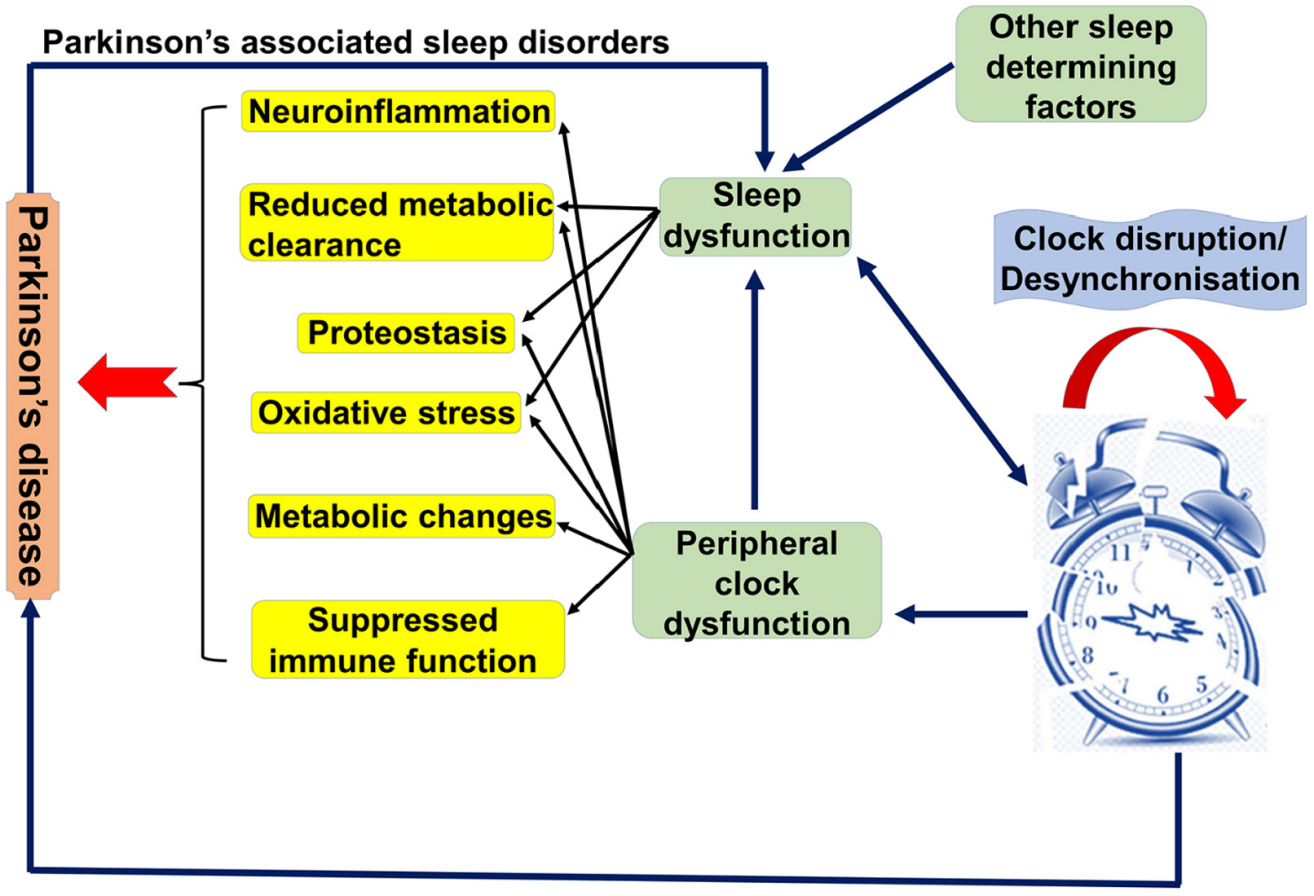
The relationship between sleep disorders and Parkinson disease.

Therefore, there is a bidirectional relationship between PD and sleep disorders, as these disorders may increase the risk of the development of PD and other neurodegenerative diseases (Figure 3).

### 7.1 Sleep disorders and α‐Syn

Sleep disorders increase the production of Aβ, which in turn promotes the expression of pro‐inflammatory cytokines in the microglial cells.^158^ This vicious cycle enhances the development and progression of neurodegeneration, including PD, and this may explain the development of dementia in the late stages of PD.^159^ Remarkably, sleep disorders increase the severity and progression of PD by enhancing release, deposition, and clearance of α‐Syn.^148^ It has been shown that sleep disorders are linked with an elevation of CSF α‐Syn in the early and prodromal phases of PD.^160^ A case‐control study that involved 46 prodromal PD patients and 169 healthy controls illustrated that CSF α‐Syn was low in PD patients compared to controls.^160^ As well, CSF α‐Syn was reported to be reduced in old Chinese subjects.^161^ A longitudinal study observed that the presence of α‐Syn in the CSF was linked with future PD risk.^162^ It has been observed in a case‐control study that plasma α‐Syn levels were increased in patients with sleep apnea due to chronic intermittent hypoxia‐induced inflammatory changes.^163^ Peripheral α‐Syn can induce PD neuropathology, as peripheral injection of α‐Syn can cause PD phenotype and α‐synucleinopathy in mice.^164^ Dos‐Santos et al.^165^ revealed that sleep‐controlling neurons are highly susceptible to the toxic effects of α‐Syn. It has been shown that α‐Syn induces alteration of membrane current, augmentation of intracellular Ca^2+^, increasing neuronal firing, and induction of neuronal cell deaths in sleep‐controlling nuclei.^165^ In a remarkable way, α‐Syn can be found in the olfactory mucosa of patients with RBD.^166^ A cross‐sectional study confirmed that olfactory dysfunction was common in patients with RBD. Therefore, nasal swabbing for α‐Syn in patients with RBD can predict the development of α‐synucleinopathy and PD.^166^ These observations give a clue regarding α‐Syn as a potential link between sleep disorders and PD neuropathology. Besides the effect of GABA on sleep control, GABA can also modulate α‐Syn release and clearance.^167^ Release of α‐Syn from glutamatergic neurons through sulfonylurea receptor type 1 is regulated by the presynaptic GABA receptor of the GABAergic neurons.^167^ Therefore, GABA agonists through modulation of α‐Syn and sleep disorders could be effective in the management of PD and related sleep disorders.

### 7.2 Sleep apnea and GABA

Furthermore, sleep apnea‐induced intermittent hypoxia can trigger PD neuropathology by increasing the development of oxidative stress and inflammation.^168^ A population case‐controlled study revealed that patients with sleep apnea were at higher PD risk compared to controls.^169^ However, the prevalence of sleep apnea is higher than that of PD, suggesting other risk factors that increase the effect of sleep apnea in the development of PD.^169^

Notably, sleep disorders promote the development of neuroinflammation by increasing the expression and release of pro‐inflammatory cytokines from brain microglial cells.^170^ An experimental study demonstrated that pre‐operative sleep disorder leads to postoperative cognitive dysfunction in aged mice due to the development of neuroinflammation.^170^ An experimental study conducted by Zhang et al. observed that dexmedetomidine restores normal sleep in rats with hepatic encephalopathy by inhibiting the development of neuroinflammation.^171^ Neuroinflammation is common in PD due to microglial over‐activation, leading to progressive neuronal injury.^172^ Thus, sleep disorder‐induced neuroinflammation could be a possible mechanistic pathologic pathway in the development of PD. A reduction in GABA levels is linked to glutamate toxicity and neuroinflammation.^173^ Therefore, GABA_A_ agonists can restore normal sleep and prevent neuroinflammation‐induced PD^174^; however, GABAA agonists can worsen sleep apnea, so they should be used with caution in these patients.

### 7.3 RBD and GABA

**FIGURE 4:**
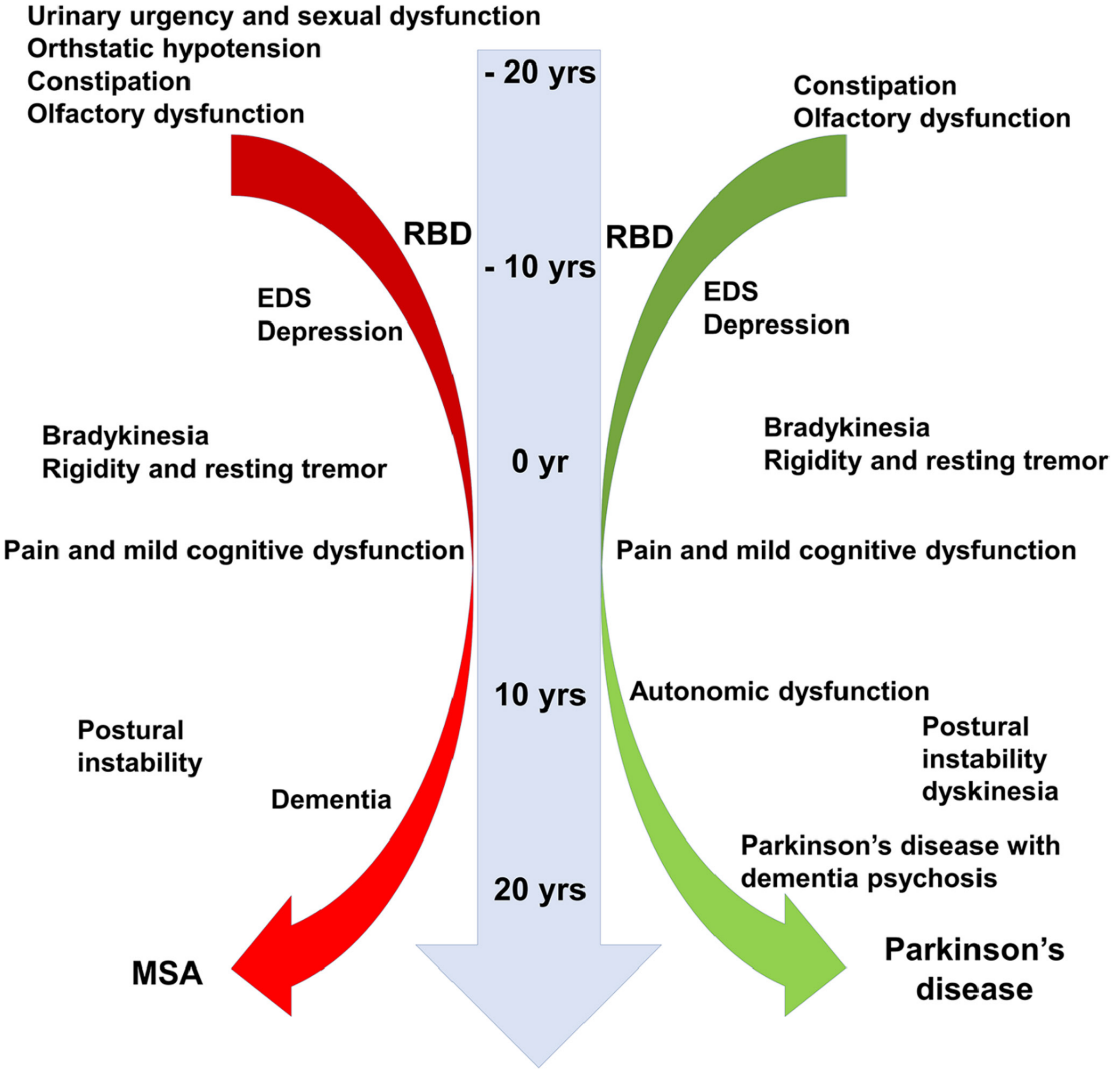
Development of Parkinson disease in relation to sleep disorders.

It has been shown that RBD pathophysiology precedes PD motor symptoms as it begins in the autonomic neurons and affects the locus coeruleus before degeneration of DNs in the SNpc.^175^ Evidence for this phenomenon is that fully developed RBD pathology is recognized at the time of PD diagnosis.^176^ Alteration in the activity of GABAergic neurons in the brainstem is associated with the development of RBD in an animal model study.^177^ Interestingly, changes due to dysbiosis disturb the gut–brain axis, which affects brain GABAergic neurons in the brainstem,^178^ leading neuroinflammation and the progression of cognitive dysfunction. GABA‐producing bacteria from the gut promote an increase in GABA in the gut, which is transported via the vagus nerve to the brain, leading to a potential imbalance of the GABA/glutamate axis with subsequent alteration of the sleep neuronal circuit and the development of sleep disorders. Notoriously, GABA can be identified in the brain before the development of α‐synucleinopathy.^179^ Thus, RBD and other sleep disorders can precede motor and most non‐motor symptoms in PD by decades (Figure 4). A cohort study followed 27 patients with RBD for at least 15 years before the development of PD dementia or other neurological diseases revealed that the development of neurological syndromes, including PD, occurred up to 50 years from the initial RBD manifestation.^179^ However, most RBD patients have α‐synucleinopathy at the time of diagnosis of PD.^180^ A cohort study that included brain autopsies from patients with RBD showed a strong correlation between RBD pathology and α‐synucleinopathy.^180^ Degeneration of neuronal pathways regulating REM sleep is associated with the burden of α‐synucleinopathy.^180^ A longitudinal study involving 61 PD patients with or without RBD at baseline followed for 4 years revealed that PD patients with RBD were at higher risk for the development of dementia.^153^

### 7.4 Glymphatic system, sleep disorders, and GABA

Indeed, the glymphatic system, which is a paravascular pathway, enhances clearance of waste products from the brain and delivery of nutrients, is highly active during sleep and prevents deposition of Aβ in AD.^181^ Extracellular α‐Syn can induce neuroinflammation and progressive neuronal cell deaths in PD. The glymphatic system removes extracellular protein metabolites and waste products during sleep.^182^ Glymphatic system function is positively correlated with delta power and negatively with beta power in the EEG recording of anesthetized mice.^183^ Disturbance of the glymphatic system by sleep disorders attenuates clearance of α‐Syn with the development of PD neuropathology.^184^ Also, the function of the glymphatic system has been revealed to be highly distorted by aging, with an increasing risk of the deposition of toxic proteins prone to intracellular accumulation.^184^ The dysfunctional glymphatic system is associated with the accumulation of α‐Syn and tau protein, leading to the propagation of neurodegenerative diseases, including AD and PD.^184^ An experimental study illustrated that the glymphatic system is extremely distorted in PD due to a deficiency of aquaporin‐4 (AQP4), leading to the progressive accumulation of α‐Syn.^185^ Supporting this notion, blockage of meningeal lymphatic drainage has been reported to exacerbate PD‐like pathology in mice by increasing the expression of mutated α‐Syn.^186^ Recently, neuroimaging evidence revealed that dysfunction of the glymphatic system is linked with the development of both PD and RBD.^187^ A case–control study involving 168 PD patients and 129 healthy controls observed that diffusion tensor image analysis along perivascular space (DTI‐ALPS) index, a reflector of glymphatic system function, was low in PD patients compared to healthy controls,^187^ suggesting dysfunction of the glymphatic system and a pathway for α‐Syn accumulation in PD. Of interest, GABA enhances the activity of the glymphatic system through modulation of the expression of AQP4, and GABA_A_ receptors are co‐localized with AQP4.^188^, ^189^ Activation of GABAergic neurons by barbiturates and propofol improves clearance mediated by the glymphatic system.^190^ Thus, enhancement of sleep patterns by GABA agonists may reduce earlier PD neuropathology by promoting the function of the glymphatic system and the build‐up of α‐Syn. Of note, aging alone or due to induced sleep disturbances reduces the glymphatic system clearance capacity with the aggregation of α‐Syn.^191^ Remarkably, the clearance capacity of the glymphatic system depends mainly on delta wave sleep during the sleep cycle.^192^ Notably, there is an inverse relationship between glymphatic system clearance capacity and the power of the delta wave.^193^ In the aging process there is dramatic reduction in the power of delta wave that may explain aging‐induced neurodegeneration, including PD.^194^

### 7.5 Chaperon‐mediated autophagy, sleep disorders, and GABA

**FIGURE 5:**
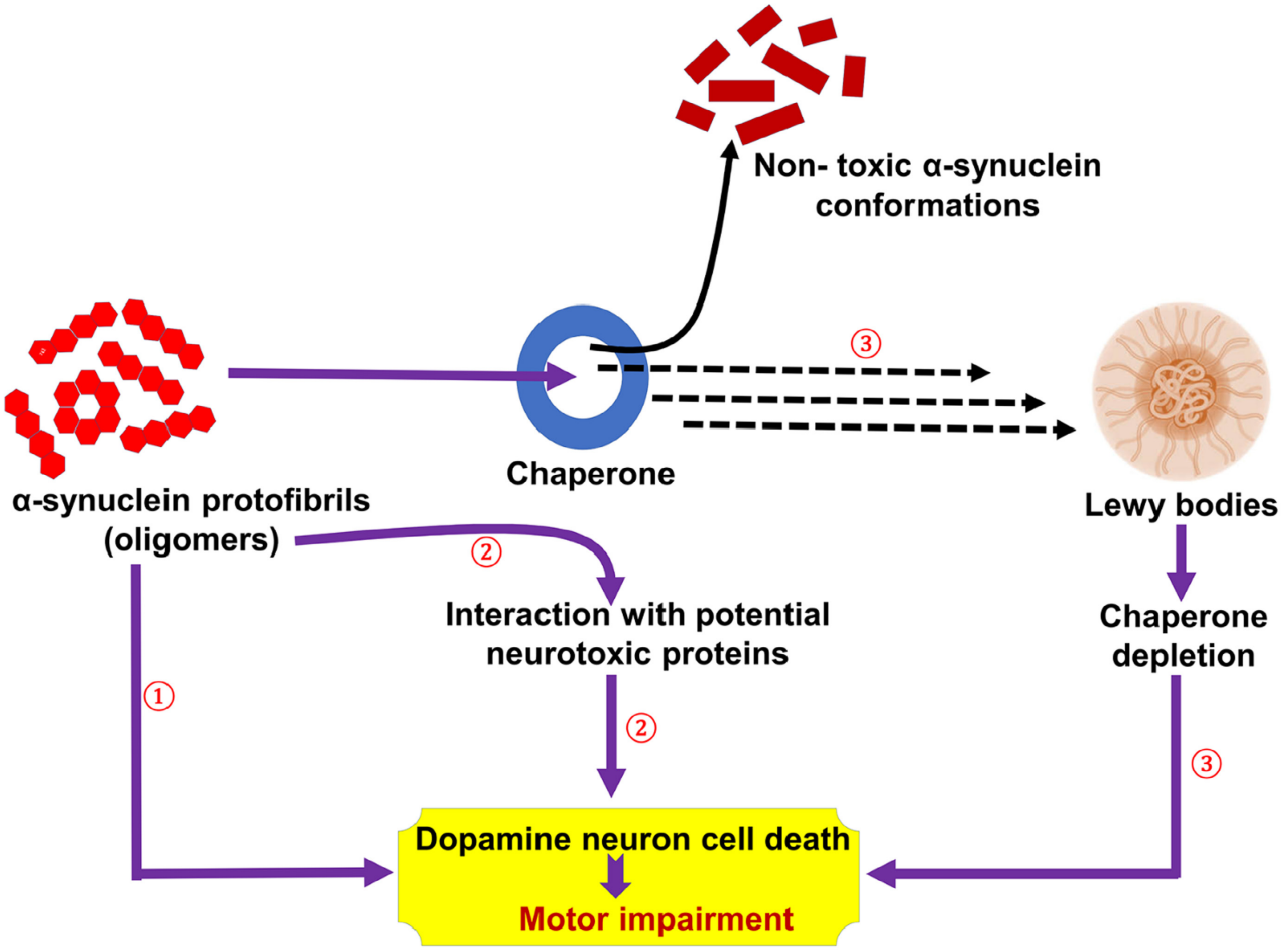
Chaperon‐mediated autophagy in Parkinson disease.

Furthermore, the aging process is linked with the diminution of the chaperon system, which prevents the accumulation of misfolded proteins.^195^ Chaperon‐mediated autophagy is involved in the degradation of intracellular cytosolic proteins.^195^ The reduction of lysosomal‐associated protein type 2A (LAP2), which acts as a receptor for chaperon‐mediated autophagy, was confirmed by an experimental study in aged mice.^195^ Chaperon‐mediated autophagy modulates the expression of neuronal transcription factors involved in neuronal survival.^196^ Dysregulation of chaperon‐mediated autophagy by α‐Syn triggers neuronal loss in PD neuropathology.^196^ In sum, chaperon‐mediated autophagy plays a critical role in preventing Lewy body formation and α‐Syn‐induced DNs injury (Figure 5). A previous study conducted by Webb et al.^197^ revealed that α‐Syn is degraded by proteasomes and autophagy. However, mutant α‐Syn blocks lysosomal membrane receptors, preventing further degradation of α‐Syn by chaperon‐mediated autophagy.^198^ Stimulation of autophagy by rapamycin promotes clearance of α‐Syn.^197^ Thus, autophagy activators could be effective in the management of PD.

Notably, autophagy disorders are linked to the development of sleep disorders.^199^ RDB induces dysregulation of autophagy, which causes neuronal injury and disruption of neuronal integrity, leading to neurodegeneration and the development of PD.^199^ Cheng et al.^200^ observed that fragmented sleep dysregulates the autophagy process. An experimental study demonstrated that the autophagy process was highly sensitive to short‐term sleep fragmentation, suggesting that dysregulation of autophagy might be the primary initiator of sleep disorders in neurodegenerative diseases like PD.^200^ Furthermore, GABAergic neurons are associated with the autophagy process.^201^ GABAergic neurons enhance autophagy activation and phagosome maturation via the activation of the GABA_A_ receptor.^201^ Of interest, autophagy is regarded as a possible link between GABAergic neuron signaling and mTOR during the neurodevelopmental process.^202^ It has been shown that GABAergic neurons promote cognitive function and reduce neuroinflammation through the induction of autophagy.^203^ In this sense, GABA activators and GABA_A_ receptor agonists can ameliorate both sleep disorders and PD neuropathology by enhancing autophagy.

### 7.6 Endoplasmic reticulum (ER) stress, sleep disorders, and GABA

It has been shown that endoplasmic reticulum (ER) stress and unfolded protein response (UPR) are linked with PD neuropathology.^204^ The accumulation of misfolded proteins in the lumen of the ER triggers the development of ER stress, with the activation of UPR as a compensatory mechanism to enhance the degradation of misfolded proteins.^205^ However, in severe ER stress, the activated cellular signaling leads to progressive neuronal injury and the development of PD.^205^ Likewise, ER stress leads to intracellular Ca^2+^ dyshomeostasis with activation of inflammasomes and autophagy.^206^ These findings implicate ER stress in the development and progression of PD neuropathology.

**FIGURE 6:**
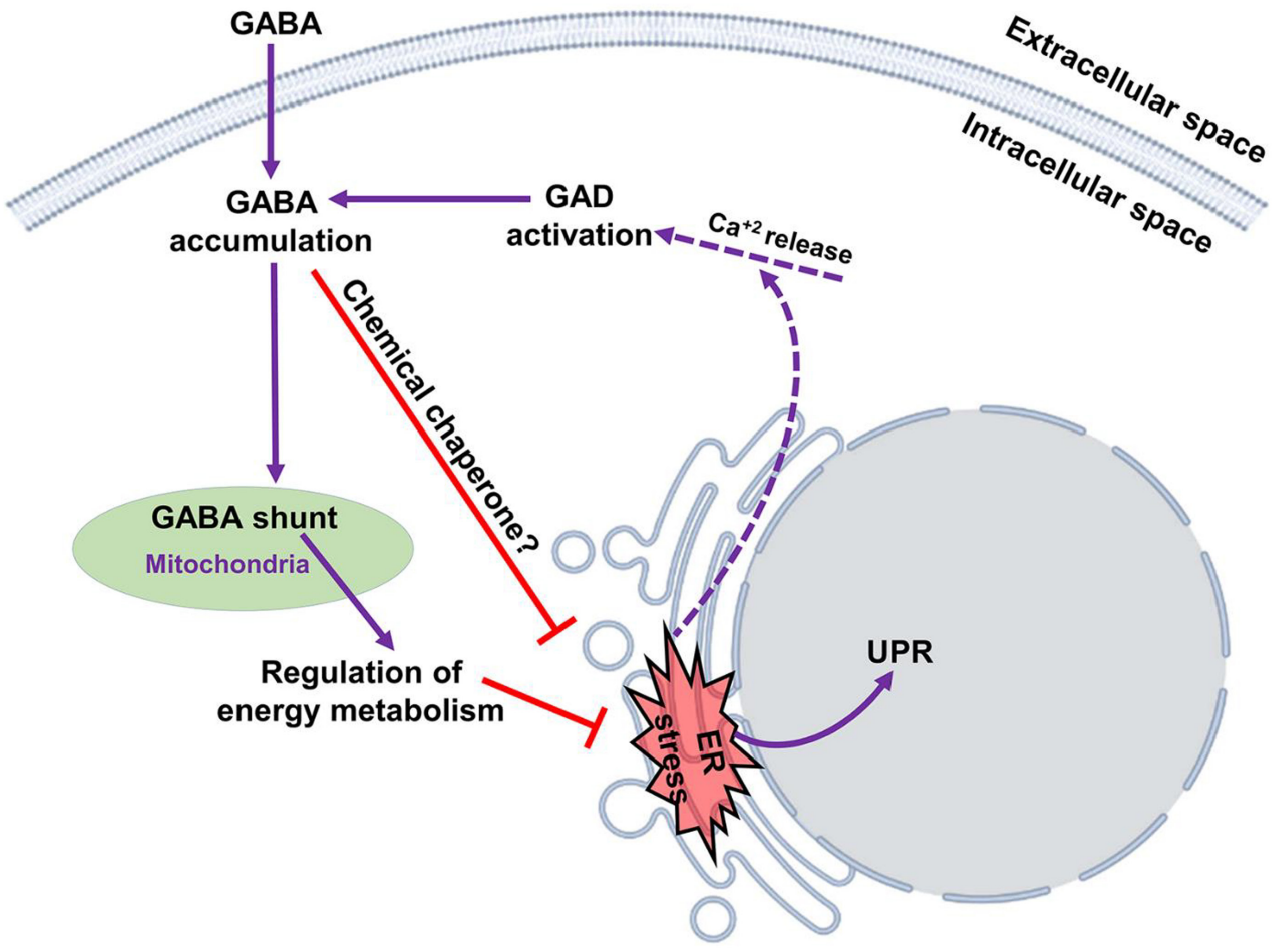
Role of GABA in endoplasmic reticulum (ER) stress.

Besides, sleep disorders like sleep fragmentation are associated with the development of ER stress.^207^ Accumulation of misfolded proteins triggers the development of ER stress, which promotes the expression of UPR.^208^ UPR enhances the expression of ER chaperon, which limits protein phosphorylation and accumulation.^208^ Normally, acute sleep deprivation triggers the expression of UPR; however, aging attenuates this response, leading to the accumulation of misfolded and pro‐apoptotic proteins that cause neuronal injury.^209^ Aging, together with sleep disorders in PD and other neurodegenerative disorders, inhibits UPR expression with further accumulation of misfolded proteins.^210^, ^211^ For example, aggregation of Aβ interferes with sleep neuronal circuits, leading to an abnormal sleep pattern with fragmented sleep in AD.^212^ In turn, poor sleep behavior enhances Aβ accumulation, causing more deterioration of AD neuropathology.^213^ The same is also true for PD, as sleep disorders and PD neuropathology promote aggregation and accumulation of α‐Syn, which drive more progressive deterioration in both PD and sleep disorders in a positive feedback loop.^160^ Remarkably, GABA regulates ER stress by controlling the expression of UPR (Figure 6).^214^ However, severe ER stress triggers the injury of GABAergic neurons in amygdala, leading to neuropsychiatric disorders including anxiety.^215^ Hence, potentiation of GABAergic neurons by GABA agonists may reduce the deleterious effects of ER stress on PD neuropathology and sleep disorders.

### 7.7 Tau protein, sleep disorders, and GABA

It has been reported that the accumulation of tau protein in PD is regarded as an early biomarker of PD‐associated dementia.^216^ It has been shown that AD biomarkers could be predictive of cognitive dysfunction in PD patients. For example, Aβ_42_ CSF level is positively correlated with cognitive impairment in PD patients.^217^ Remarkably, a low Aβ_42_ level in the CSF is linked with the future development of hallucinations and illusions.^218^ Notably, aggregation of tau protein is not limited to AD but is also involved in PD neuropathology as a disorder of tau protein was observed in about 50% of sporadic PD.^219^ Tau hyperphosphorylation and interaction with α‐Syn contribute to neuronal injury in PD.^219^ Tau protein is closely distributed with α‐Syn, which is positively correlated with cognitive dysfunction.^220^ Both the synthesis and release of α‐Syn and tau proteins are augmented by sleep deprivation.^220^, ^221^ In addition, the α‐Syn CSF level is not affected by diurnal variation,^222^ but tau protein CSF level is higher during night and dark environments.^223^ The link between tau protein and Aβ sleep disorders is complex. Experimental administration of Aβ_25‐35_ in mice induces a reduction in NREM sleep and increases wakefulness.^224^ Besides, expression of tau protein and orexin A was increased in the brain tissue of AD mice compared to control mice.^224^ In vitro studies demonstrated that tau protein, orexin A, and adenosine were increased in cell lines exposure to Aβ_25‐35_ compared to normal cell lines.^224^ These observations suggest that tau protein and Aβ lead to sleep disorders through increased expression of excitatory orexin A and adenosine. However, orexin CSF levels are reduced in PD patients due to the progressive loss of orexin during PD neuropathology.^225^ Therefore, PD patients experience narcolepsy‐like symptoms due to the degeneration of orexinergic neurons.^226^ Furthermore, tau protein pathology induces sleep disorders through dysregulation of GABAergic neurons.^227^ Tau pathology in an experimental study had been confirmed to cause severe synaptic dysfunction and memory deficits in mice through impairment of GABAergic neurons.^227^ Therefore, enhancement of the activity of GABAergic neurons by GABA activators may reduce tau pathology‐induced cognitive deficits and sleep disorders.^54^ The effects of GABA activator benzodiazepines on tau pathology and neurodegenerations are controversial. For example, an experimental study conducted by Whittington et al.^228^ showed that administration of benzodiazepine midazolam augmented tau protein phosphorylation in mice. In contrast, a new benzodiazepine remimazolam delays neurodegeneration in mice by reducing tau phosphorylation.^229^ However, a review performed by Al‐Kuraishy and his colleagues illustrated that benzodiazepine use in AD was associated with beneficial rather than detrimental effects.^35^ Therefore, GABA activators may play a crucial role in preventing tau pathology‐induced sleep disorders in neurodegenerative disorders, including PD.

### 7.8 Brain‐derived neurotrophic factor, sleep disorders, and GABA

It has been observed that brain‐derived neurotrophic factor (BDNF) has a neuroprotective effect against the development and progression of PD by preserving the survival of DNs in the SNpc.^230^ According to animal model and human studies, BDNF levels were reduced in PD.^230^ BDNF exerts its biological effects through stimulation of surface receptor tyrosine kinase (Trk) and the p75 neurotrophin receptor (p75NTR).^231^ BDNF has a potent anti‐apoptotic effect, preventing the degeneration of DNs in the SNpc; thus, reduction of BDNF promotes PD neuropathology. A case‐control study included 47 PD patients and 23 healthy controls showed that BDNF serum levels were reduced in early PD patients compared to healthy controls.^232^ A systematic review and meta‐analysis revealed that BDNF serum levels were reduced and correlated with motor severity in PD patients.^233^ BDNF is regulated by the effect of miRNA‐7, which reduces PD neuropathology by increasing expression of BDNF and reducing the generation of α‐synuclein in a rat PD model.^233^ Virachit et al.^234^ observed that BDNF serum levels were increased in late PD as a compensatory mechanism to mitigate inflammatory changes. Besides, experimental and clinical findings suggest a protective role of BDNF against sleep disorders. A case‐control study that involved patients with sleep disorders and healthy controls illustrated that BDNF serum level was associated with the progression of sleep disorders.^235^ BDNF serum level is increased in patients with sleep apnea to mitigate the associated neurocognitive dysfunction.^236^ Different studies have reported that BDNF has a protective effect on the GABAergic neurons; it increases activity and maturation of these neurons.^237^, ^238^ Reduction of BDNF by aging the process induces degeneration of hippocampal GABAergic neurons with a diminution of GABAergic neurons neuroplasticity.^239^ These findings propose that a reduction of BDNF in PD promotes the development of GABAergic neuron dysfunction and the initiation of sleep disorders.

**FIGURE 7:**
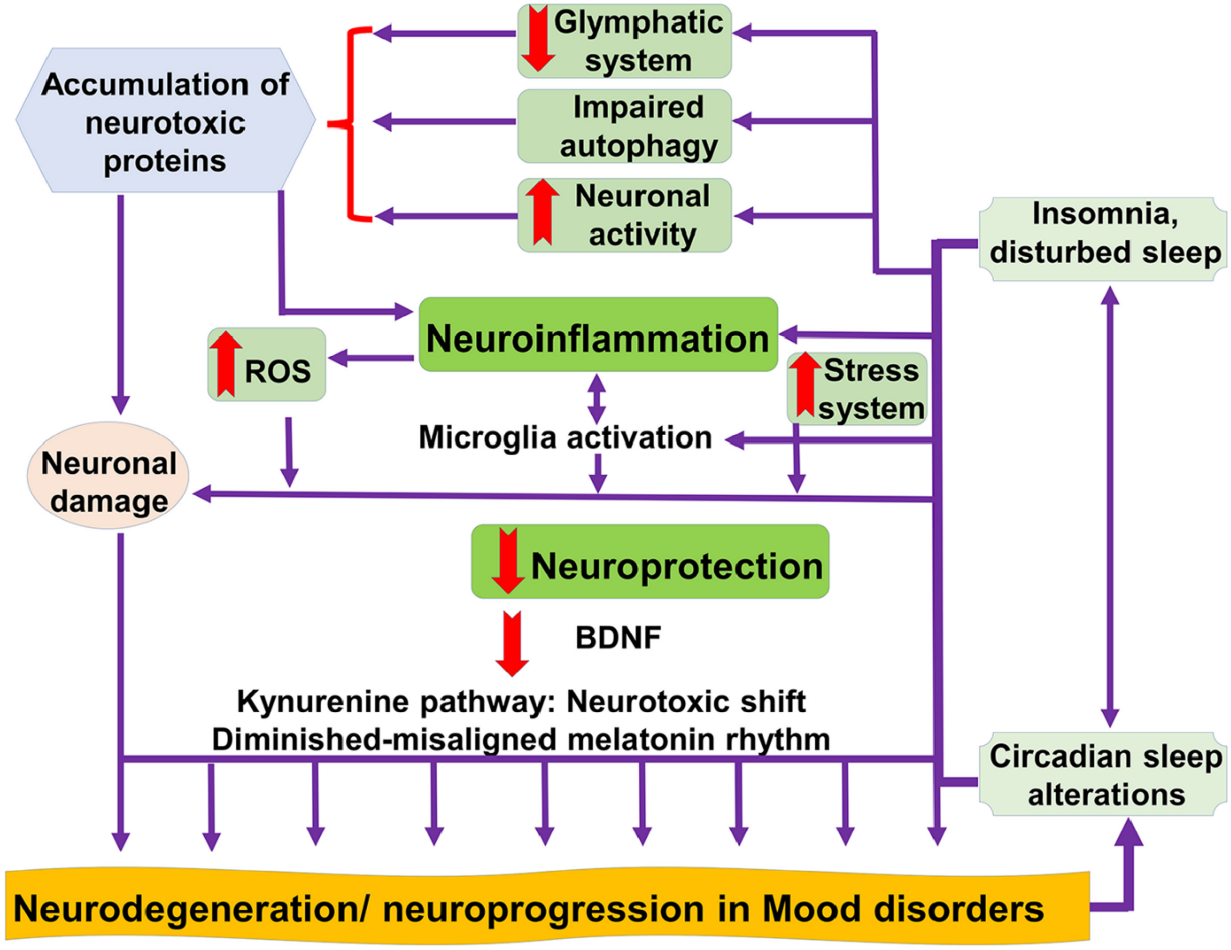
Mechanistic role of sleep disorders in the development of Parkinson disease.

Taken together, non‐motor symptoms, mainly insomnia and disturbed sleep, promote neuroinflammation with the accumulation of neurotoxic proteins due to defects in autophagy, endoplasmic reticulum (ER) stress, and the glymphatic system (Figure 7).

The present review has many limitations, including the paucity of prospective studies, and most findings are taken from observational and preclinical studies. GABA involvement in the pathogenesis of PD has been recently discussed in recent studies. However, the underlying mechanistic role of GABA in PD was not confirmed clinically. Therefore, future perspective studies regarding the use of GABA agonists in the management of PD are recommended to observe their differential effects on motor and non‐motor symptoms.

## 8 CONCLUSION

PD is a progressive neurodegenerative disease due to DNs loss in the SNpc. The primary features of PD are motor symptoms like bradykinesia, resting tremor, and rigidity, and non‐motor symptoms such as sleep disorders. PD symptoms develop in reaction to disturbances of diverse neurotransmitters, including GABA. Sleep disorders are linked to dysfunction of the GABA pathway. Sleep disorders are connected with a decrease in brain GABA levels. In addition, the lessening of prefrontal GABA is associated with memory and cognitive dysfunction. GABA has an imperative role in normal sleep, and dysfunction of GABAergic neurons is associated with the development of sleep disorders. PD neuropathology is associated with sleep disorders in about 98% of cases. RBD is commonly developed in the early stages of PD and adversely affects the cognitive function of PD patients. Furthermore, PD‐related disorders like rigidity and nocturia can also affect sleep patterns, leading to fragmented sleep, which may also affect PD neuropathology. Consequently, there is a bidirectional interaction between PD and sleep disorders, as these disorders may increase the risk of the development of PD and other neurodegenerative diseases. Sleep disorders augment the severity and progression of PD by enhancing α‐synucleinopathy. GABA can also adapt α‐Syn release and clearance. The release of α‐Syn is regulated by the presynaptic GABA receptor of the GABAergic neurons. In sum, non‐motor symptoms, mainly insomnia and disturbed sleep, promote neuroinflammation with the accumulation of neurotoxic proteins due to defects in autophagy, ER stress, and the glymphatic system. Thus, GABA agonists via modulation of α‐Syn and sleep disorders might be effective in the management of PD and related sleep disorders. In this state, preclinical and clinical studies are warranted in this regard to elucidate the causal relationship between PD and sleep disorders regarding the role of GABA.

## AUTHOR CONTRIBUTIONS

The study was conducted by Hayder M. Al‐Kuraishy, Yaser Hosny Ali Elewa, Ammar AL‐Farga, Faisal Aqlan, Mahmoud Hosny Zahran, and Gaber El‐Saber Batiha. The manuscript was written by Hayder M. Al‐Kuraishy and Mahmoud Hosny Zahran, with edits from Yaser Hosny Ali Elewa, Ali I. Al‐Gareeb, Ammar AL‐Farga, Faisal Aqlan and Ali K. Albuhadily and a review by Gaber El‐Saber Batiha.

## CONFLICT OF INTEREST STATEMENT

All authors declare that there are no conflicts of interest to disclose.

## Data Availability

The data that support the findings of this study are available from the corresponding author upon reasonable request.
